# An Optimal Image-Based Method for Identification of Acoustic Emission (AE) Sources in Plate-Like Structures Using a Lead Zirconium Titanate (PZT) Sensor Array

**DOI:** 10.3390/s18020631

**Published:** 2018-02-21

**Authors:** Gang Yan, Li Zhou

**Affiliations:** State Key Laboratory of Mechanics and Control of Mechanical Structures, College of Aerospace Engineering, Nanjing University of Aeronautics and Astronautics, Nanjing 210016, China; lzhou@nuaa.edu.cn

**Keywords:** AE source identification, reverse-time f-k migration, image processing, minimum Shannon entropy, artificial bee colony algorithm

## Abstract

This paper proposes an innovative method for identifying the locations of multiple simultaneous acoustic emission (AE) events in plate-like structures from the view of image processing. By using a linear lead zirconium titanate (PZT) sensor array to record the AE wave signals, a reverse-time frequency-wavenumber (f-k) migration is employed to produce images displaying the locations of AE sources by back-propagating the AE waves. Lamb wave theory is included in the f-k migration to consider the dispersive property of the AE waves. Since the exact occurrence time of the AE events is usually unknown when recording the AE wave signals, a heuristic artificial bee colony (ABC) algorithm combined with an optimal criterion using minimum Shannon entropy is used to find the image with the identified AE source locations and occurrence time that mostly approximate the actual ones. Experimental studies on an aluminum plate with AE events simulated by PZT actuators are performed to validate the applicability and effectiveness of the proposed optimal image-based AE source identification method.

## 1. Introduction

Recently, with the development of novel sensing technology, there is increasing needs to develop in-situ structural health monitoring system (SHMS) to monitor the integrity of critical structures such as airplanes and detect their damages as early as possible to prevent catastrophic failure. Usually, for a specific SHMS, depending on its configuration there are two kinds of working modes: active monitoring and passive monitoring [1,2]. Active monitoring uses actuators to excite diagnostic signals into the structure and uses sensors to measure the responses to infer damage states, while passive monitoring uses only sensors to record structural responses or signals released by damages. This study considers passive monitoring of acoustic emission (AE) waves for plate-like structures to determine the locations of their sources where possible damages may exist.

Traditional AE source localization methods are based on signal processing. Most of these localization algorithms developed utilize time information extracted from the AE wave signals recorded by different sensors. Combined with the known velocity of AE waves and the distance between the sensors, the location of the AE source can be triangulated or calculated by solving a set of nonlinear equations [3,4,5,6,7,8]. The time information is usually extracted by relatively simple processing methods, such as threshold crossing [9], cross-correlation [10] and statistical Akaike information criterion (AIC) [11,12]. Recently, with the development of time-frequency signal processing, the continuous wavelet transform (CWT) has been widely used for the time-frequency representation of transient AE waves and extraction of useful time information at each local frequency for AE source localization [4,5,13,14]. Besides, other advanced signal processing techniques, such as warped Fourier transform (WFT) [15], Hilbert transform (HT) [16], short time Fourier transform (STFT) [17], have been also employed for processing the AE wave signals. In addition, probabilistic and statistical approaches, which can characterize the AE source with consideration of various uncertainties, in particular uncertainties in extracted time information and wave velocities, have received more and more attention in AE source identification. These approaches includes extended Kalman filter (EKF) [18], unscented Kalman filter (UKF) [15], particle filter (PF) [19] and Bayesian methods which are realized by Monte Carlo simulations [20,21,22]. In addition, machine learning approaches, such as artificial neural network (ANN) and support vector machine (SVM), have also been employed for AE source localization [23,24,25,26]. Results have demonstrated that ANN and SVM are promising ways to localize AE sources; however, they require a large amount of data sets for training, impeding their applications in practical use. Another big problem for these aforementioned signal processing-based approaches is that, since the AE wave signals generated by multiple simultaneous AE events are difficult to be clearly separated, the developed localization methods mentioned above lack the capability to identify the locations of multiple simultaneous AE events.

This study aims to develop a new method to identify the locations of multiple simultaneous AE events in plate-like structures. Unlike previous work on triangulation of AE source using signal processing method, the method developed in this study is based on an imaging algorithm and an optimal image processing method. The rest of this paper is structured as follows. Section 2 describes the image-based AE source identification method. A reverse-time frequency-wavenumber (f-k) migration is employed to produce image displaying the locations of AE sources by back-propagating the sensor-received AE wave signals. Lamb wave theory is included in the f-k migration to consider the dispersive nature of the AE wave signals. Since the exact occurrence time of the AE events is usually unknown when recording the AE wave signals, in Section 3 a heuristic artificial bee colony (ABC) algorithm combined with an optimal criterion using minimum Shannon entropy is given to determine the image with the identified AE source locations and occurrence time that mostly approximate the actual ones. In Section 4, experimental studies on an aluminum plate with AE events simulated by lead zirconium titanate (PZT) actuators are performed to verify the proposed method. Finally, concluding remarks are given in Section 5.

## 2. Reverse-Time F-K Migration for Imaging AE Sources

### 2.1. Reverse-Time F-K Migration

**Figure 1:**
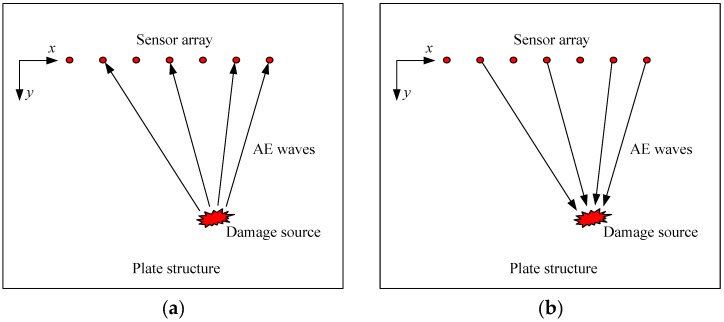
Illustration of imaging AE source by migration: (**a**) forward propagation (**b**) back propagation.

In structural health monitoring, PZT transducers are widely used to be embedded in or surface mounted on plate-like structures to act as actuators or sensors according to the piezoelectric effect and reverse piezoelectric effect of the PZT materials [27]. Figure 1 shows an illustration of a plate-like structure deployed with a linear PZT transducer array. In this study, we put our attention on AE events, thus these transducers act as sensors in a passive mode. When damages occur in the plate, elastic waves, namely AE waves, are emitted with the release of strain energy. These AE waves propagate in the structure and encounter the sensor array, the sensor array is then triggered to record the AE waves. A reverse-time f-k migration is employed to back-propagate these recorded AE waves to their sources and generate an image containing spatial information about the locations of the damage sources.

Migration, which is originally developed in geophysical prospecting to find the underground reflectors, mainly consists of two basic steps, i.e. extrapolation and imaging [28]. Extrapolation is to treat the recorded wavefield as an excitation and reconstruct the spatial wavefield on the vertical section of the earth. This is the process of back-propagating the wavefield towards the reflectors. Imaging is to use an appropriate imaging condition to build and display the strength and locations of the reflectors by extracting a variable from the extrapolated wavefield. As illustrated in Figure 1, the configuration of the plate with a linear sensor array is an analog to the vertical section of the earth, thus migration is employed for imaging the damage sources which are similar to the reflectors in geophysical prospecting.

In the Cartesian coordinate system illustrated in Figure 1, we denote the AE wavefield propagating in the plate structure as w(x,y,t). By applying 2-D Fourier transform (FT) to w(x,y,t) with respect to *t* and *x*,

(1) $$w(k_{x},y,\omega )=\int_{-\infty }^{\infty }\int_{-\infty }^{\infty }w(x,y,t)e^{i(k_{x}x-\omega t)}dxdt$$

The equation which describes the wave propagation in the f-k domain can be written as [29]

(2) $$\frac{\partial^{2}w(k_{x},y,\omega )}{\partial y^{2}}=-k_{y}^{2}w(k_{x},y,\omega )$$

in which ω is the circular frequency, *k* is the wavenumber, $k_{x}$ and $k_{y}$ are the components of k in *x*- and *y*-directions, respectively.

As illustrated in Figure 1, only the upgoing wave from the damages to the sensor array is considered and with omission of the term of harmonic wave $e^{i\omega t}$, the solution of Equation (2) can be written as

(3) $$w(k_{x},y,\omega )=Ce^{ik_{y}y}$$

in which *C* is a constant that could be determined by the boundary condition.

Suppose the sensor array is in line with the abscissa axis of the coordinate system where y=0, then the constant *C* can be determined as

(4) $$C=w(k_{x},0,\omega )$$

which is the 2-D FT of the recorded AE waves w(x,y,t) by the sensor array. Thus Equation (3) can be written as

(5) $$w(k_{x},y,\omega )=w(k_{x},0,\omega )e^{ik_{y}y}$$

By applying inverse 2-D FT to Equation (5) with respect to $k_{x}$ and ω, it yields

(6) $$w(x,y,t)=\frac{1}{4\pi^{2}}\int_{-\infty }^{\infty }\int_{-\infty }^{\infty }w(k_{x},0,\omega )e^{-i(k_{x}x-k_{y}y-\omega t)}dk_{x}d\omega$$

Equation (6) is an important formulation for the reverse-time f-k migration in this study. It back-propagates the AE waves recorded by the sensor array to their sources, namely the damages.

Then, imaging condition should be applied to the back-propagating AE waves to determine when and where they should stop to illuminate the damages [28]. In this study, the exploding imaging condition is used to visualize the damage locations. The exploding image condition considers the wave sources exist at *t* = 0, i.e.

(7) $$w(x,y,0)=\frac{1}{4\pi^{2}}\int_{-\infty }^{\infty }\int_{-\infty }^{\infty }w(k_{x},0,\omega )e^{-i(k_{x}x-k_{y}y)}dk_{x}d\omega$$

Note that, Equation (7) is not an inverse 2-D FT. To calculate it using FT and its fast algorithm, we need to map $w(k_{x},\omega )$ into $w(k_{x},k_{y})$ (here w(x,y,0) and $w(k_{x},0,\omega )$ are written as w(x,y) and $w(k_{x},\omega )$ for simplicity).

Unlike the non-dispersive waves used in geophysics, AE waves in plate-like structures are dispersive. According to the group velocity dispersion relationship [30],

(8) $$c_{g}=\frac{d\omega }{dk}=\frac{d\omega }{d(\sqrt{k_{x}^{2}+k_{y}^{2}})}$$

in which $c_{g}$ is the group velocity of the waves, Equation (7) can be written as

(9) $$w(x,y)=\frac{1}{4\pi^{2}}\int_{-\infty }^{\infty }\int_{-\infty }^{\infty }w(k_{x},\omega )e^{-i(k_{x}x-k_{y}y)}\frac{c_{g}k_{y}}{\sqrt{k_{x}^{2}+k_{y}^{2}}}dk_{x}dk_{y}$$

In addition, by mapping $w(k_{x},\omega )$ into $w(k_{x},k_{y})$ through the phase velocity dispersion relationship

(10) $$k_{y}=\sqrt{{\left(\omega /c_{p}\right)}^{2}-k_{x}^{2}}$$

in which $k=\omega /c_{p}$, $c_{p}$ is the phase velocity of the waves, Equation (9) becomes

(11) $$w(x,y)=\frac{1}{4\pi^{2}}\int_{-\infty }^{\infty }\int_{-\infty }^{\infty }w(k_{x},k_{y})\frac{c_{g}k_{y}}{\sqrt{k_{x}^{2}+k_{y}^{2}}}e^{-i(k_{x}x-k_{y}y)}dk_{x}dk_{y}$$

After the interpolation of $w(k_{x},\omega )$ to $w(k_{x},k_{y})$ by using *sinc* function in this study, Equation (11) can be solved by applying inverse 2-D FT. By using the fast algorithm of FT, Equation (11) could be conveniently solved with very high computational efficiency.

### 2.2. Lamb Wave Theory

The dispersive waves propagating in plate-like structures in directions parallel to the plate surfaces are defined as Lamb waves or guided elastic waves [30]. There are two groups of Lamb waves, symmetric and antisymmetric, that satisfy the wave equations and surface boundary conditions and each can propagate independently of the other. While the damage-induced AE waves in plate structure consists of extensional waves (symmetric Lamb waves) and flexural waves (antisymmetric Lamb waves), their dispersive nature should be included in the migration algorithm.

In three-dimensional elastodynamics, Lamb waves in an isotropic plate with thickness *h* can be characterized by Rayleigh-Lamb equations as [30]

(12) $$\frac{\tan(qh)}{\tan(ph)}=-\frac{4k^{2}pq}{{\left(q^{2}-k^{2}\right)}^{2}}$$

for symmetric modes, and

(13) $$\frac{\tan(qh)}{\tan(ph)}=-\frac{{\left(q^{2}-k^{2}\right)}^{2}}{4k^{2}pq}$$

for antisymmetric modes, in which $p^{2}=\frac{\omega^{2}}{c_{d}^{2}}-k^{2}$, $q^{2}=\frac{\omega^{2}}{c_{s}^{2}}-k^{2}$, $c_{d}=\sqrt{\frac{E}{2\left(1+v\right)\rho }}$ is the velocity of longitudinal wave and $c_{s}=\sqrt{\frac{E}{2\left(1+v\right)\rho }}$ is the velocity of shear wave, respectively, E is Young’s modulus, v is Poisson ratio and ρ is density.

Since solving the above two transcendental equations is not trivial, modeling of the dispersion behavior based on plate theories is adopted due to their computational efficiency. In the following experimental study, most of the wave components in the AE waves belong to the fundamental flexural wave, which is equivalent to A0 mode antisymmetric Lamb wave, thus this study only considers the fundamental flexural wave to image the AE sources. Mindlin plate theory is used to establish the f-k relationship of the fundamental flexural wave [31]. The wavenumber *k* of the fundamental flexural wave modeled by Mindlin theory can be given as

(14) $$k(\omega )=\sqrt{\frac{\omega^{2}}{2\kappa^{2}c_{s}^{2}}+\sqrt{{\left[\frac{\omega^{2}}{2\kappa^{2}c_{s}^{2}}\right]}^{2}+\frac{6\left(1-v\right)\omega^{2}}{h^{2}c_{s}^{2}}}}$$

in which $\kappa^{2}=\pi^{2}/12$ is the shear correction coefficient. If the AE waves contain fundamental extensional wave which is equivalent to S0 mode symmetric Lamb wave, Kane-Mindlin plate theory [32] can be used to model the dispersive relationship and embedded into the reverse-time f-k migration algorithm in a similar way.

## 3. Optimization of the AE Image

### 3.1. Shannon Entropy of Re-Focused Image

The imaging condition used in the reverse-time f-k migration in Section 2 assumes that the start point of the recorded AE wave signals is exact the occurrence time of the AE events. Nonetheless, in real applications, the occurrence time of the AE events is usually unknown and cannot be determined directly from the AE wave signals recorded by the sensor array. This means that the AE waves may not be back-propagated to their exact source locations by migration if the signal length of the AE waves is not consistent with the right occurrence time. Thus, an important problem should be solved for applying the reverse-time f-k migration method is to determine the occurrence time and the image contains information of the source locations that mostly approximates the actual ones. Derveaux et al. proposed an optimal method with a time-reversal technique to determine the time when the time-reversed wavefield can focus on the damages [33]. The basic idea of their study is employed in this study, an optimal criterion using minimum entropy principle is adopted to achieve the objective.

When the AE waves focus back to the damage sources, it is expected to produce a re-focused image with sharp peaks near the actual source locations and significantly diminish elsewhere. In image processing area, sparsity norms are usually used as a measure to characterize focused images, which may be small in that case and large otherwise. In this study, Shannnon entropy, which is defined in information theory as [34]

(15) $$S(Z)=-\sum_{z\in ℵ}p(z)\log_{2}p(z)$$

is employed as the norm of measure. In Equation (15), p(z) is the probability density function of random variable Z.

For f-k migration, after the reversed AE waves is obtained, at every discrete pixel point (*x_m_*, *y_n_*) (*m* = 1 to *M*, *n* = 1 to *N*, *M* and *N* are the number of pixels in horizontal and vertical directions, respectively), an image displaying *w*(*x*,*y*) in a discrete form *w*(*x_m_*, *y_n_*) can be produced. In this study, the amplitude of the wavefield *w*(*x*,*y*) is normalized by its maximum as $\tilde{w}(x,y)=w(x,y)/\max(w(x,y))$ and the produced image is designated as ${\tilde{w}}_{mn}$ for convenience. For a pixelated image, the entropy by Shannon’s definition is a measure of the sparsity of the histogram of the grey levels of the image [33]. From Equation (15), the Shannon entropy of the image ${\tilde{w}}_{mn}$ can be defined by

(16) $$S({\tilde{w}}_{mn})=-\sum_{k=0}^{N_{c}-1}\left(\frac{h_{k}}{M\cdot N}\right)\log_{2}\left(\frac{h_{k}}{M\cdot N}\right)$$

where *h_k_* is the histogram of grey levels of the image which can be determined by counting the number of pixels contained in each grey level, *N_c_* is the number of grey levels, M⋅N is the total number of pixels. The entropy quantifies the amount of information needed to encode an image and it penalizes back propagated fields that have a lot of speckles [33].

### 3.2. Artificial Bee Colony Algorithm

The ABC algorithm is proposed by Karaboga and Bastur with the inspiration by the natural behavior of honey bees when foraging (seeking high quality food sources) [35,36]. In this study, finding the optimal image with minimum Shannon entropy and determining the occurrence time of AE events is formulated as finding the best food source in the ABC algorithm. By mimicking the behaviors of different types of bees involved in the foraging process, there are three search phases in the standard ABC algorithm: employed bee search, onlooker bee search and scout bee search. For determining the occurrence time of AE events and the optimal image by using ABC algorithm, the detailed steps are presented as follows:

(1) Initialization. The first step of the ABC algorithm is to generate an initial set of possible food sources, i.e. possible occurrence times in this study. With specified ranges, the generic *l*th possible solution of occurrence time $t_{o}^{l}$ can be randomly generated as

(17) $$t_{o}^{l}=t_{o}^{\min}+\mu \left(t_{o}^{\max}-t_{o}^{\min}\right)$$

where *l* = 1,2, …,*np*, $t_{o}^{\min}$ and $t_{o}^{\max}$ are the lower and upper bounds of occurrence time, μ is a random number between −1 and 1. From these initial *np* occurrence times, those *nf* ones that have the smallest objective values are chosen to form the initial population (*nf* is set to be *np*/2 in this study).

(2) Employed bee search. A first stage search for updated solutions in the neighborhood is conducted by starting from the initial set of solutions. This is very similar to the employ bees’ task in nature, trying to find better food sources by a neighborhood search. In this search phase, an updating strategy is designed to generate a new candidate for every solution at each iterative step. Here in this study, the new candidate solution $t_{o}^{c}$ is generated as:

(18) $$t_{o}^{c}=t_{o}^{l}+\mu \left(t_{o}^{l}-t_{o}^{r}\right)$$

with *r* = 1,2, …, *nf* and l≠r. The fitness function is defined as

(19) $$fit\left(t_{o}\right)=\frac{1}{1+obj\left(t_{o}\right)}$$

where $obj\left(t_{o}\right)$ is the objective function defined in Equation (16), i.e. the Shannon entropy value of the image produced by reverse-time f-k migration with AE wave signals starting at assumed occurrence time $t_{o}$. If the fitness value of the candidate is better than that of the previous solution, then the solution will be updated; otherwise, the previous solution is kept.

(3) Onlooker bee search. After employed bee phase have identified all the possible candidate solutions, onlooker bee phase is performed to select food sources with a higher probability values. Here in this study, the probability of selection is proportional to the fitness and the probability associated with the *l*th candidate is defined as

(20) $$prob_{l}=\frac{fit\left(t_{o}^{l}\right)}{\sum_{r}^{nf}fit\left(t_{o}^{r}\right)}$$

After selecting a food source by the roulette wheel selection strategy, a new *l*th solution can be obtained by Equation (18) and a comparison of its fitness and that of the current one is conducted. The solution which has a larger fitness value is selected as the representative of the *l*th solution and the new set of *nf* possible solutions represents the starting point of the next iteration. To determine the current best solution, the solution with the largest fitness value is found at current iteration and compared with the previous ones.

(4) Scout bee search. During the search iterations, for some possible solutions, for instance, the *l*th solution, it might happen that they are not improved for a given number of iterations, while other solutions have continuous improvement of their fitness. Usually, this means that the *l*th solution reaches a local optimum of the objective function. When this happens, the stagnated solution should be replaced by a new solution which is generated by using Equation (17). The design of such a scout bee search can enhance the algorithm with an improved global search capacity, providing a way to exit stagnation points.

**Figure 2:**
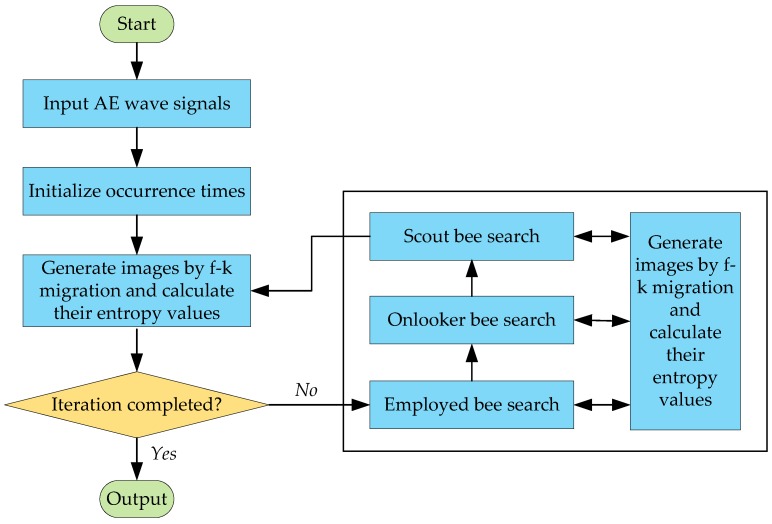
Flow chart of the proposed method.

Figure 2 shows the flow chart of the proposed method for AE source identification using reverse-time f-k migration and ABC algorithm. After input of the AE wave signals, the identification process is initiated by random generation of a set of individuals (occurrence times) and is updated by the operation of bee search phases, proceeding toward a global optimum target. In the updating process, AE wave signals from the occurrence time to the end are processed by reverse-time f-k migration to form images and their entropy values are evaluated by Equation (16) as the objective function values used for the ABC algorithm. After the final iteration reaches, the global optimal estimation of the occurrence time is obtained and the migration algorithm is performed again to finally image the AE sources.

## 4. Experimental Study

### 4.1. Experimental Set-Up

**Figure 3:**
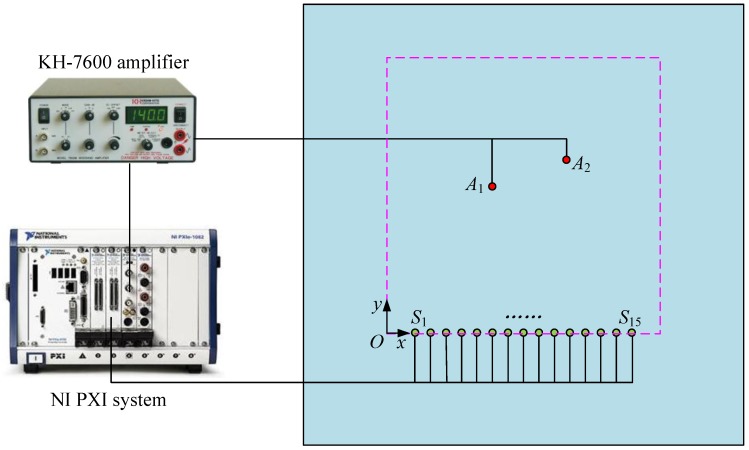
Test setup for imaging of damage sources.

**Figure 4:**
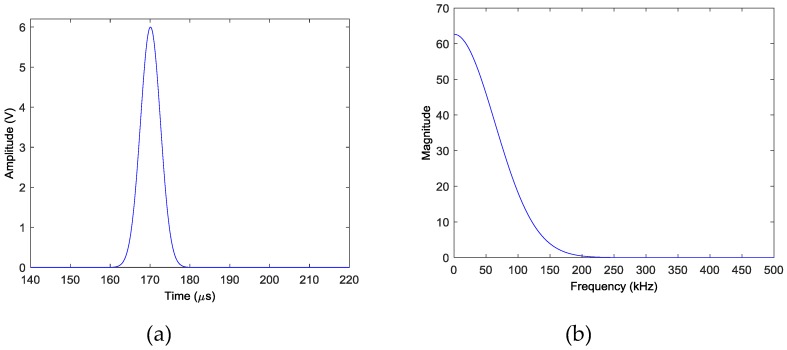
Impulse excitation: (**a**) time trace (**b**) frequency spectrum.

Experimental studies on an aluminum plate are conducted to verify the effectiveness of the proposed method. The experimental set-up comprises an aluminum plate and a National Instrument PXI system incorporated with a PXI-5441 signal generator and two PXI-5105 digitizers and a KH-7600 amplifier. The aluminum plate has dimensions of 1000 mm × 1000 mm × 2 mm and its nominal properties are as follows: elastic modulus E = 73 GPa, Poisson ratio v = 0.32 and density ρ = 2790 kg/m^3^. A linear sensor array containing 15 PZT transducers, denoted by *S*_1_ to *S*_15_, with thickness of 1 mm and diameter of 10 mm are mounted on the surface of the plate. These sensors are connected to the data acquisition channels of the digitizers to monitor the damage events and collect the AE wave signals. Unfortunately, *S*_4_, *S*_12_ and *S*_13_ malfunction in the later test, so only the 12 normal sensors are used for damage imaging. Another 2 PZT transducers with the same size and material, denoted by *A*_1_ and *A*_2_, are also mounted on the surface of the plate to act as actuators to excite AE waves to simulate the damage events. The aluminum plate and sensor placement is illustrated in Figure 3 (not in scale). The origin of the Cartesian coordinate system is set at the left of *S*_1_, with distance of 75 mm. The coordinates of the center locations of sensors *S*_1_ and *S*_15_ are (75, 0) mm and (425, 0) mm, respectively. The remaining 13 sensors are uniformly distributed between *S*_1_ and *S*_15_ with spacing of 25 mm. The coordinates of the center locations of actuators *A*_1_ and *A*_2_ are (190, 250) mm and (295, 300) mm, respectively. During the experiment, a wideband impulse excitation as shown in Figure 4 is generated by the signal generator, amplified by the amplifier and applied the actuators to simulate the burst of AE event. The amplitude of the signal output by the signal generator is 6 V and the gain of the amplifier is 28 dB. The simulated AE waves are then received by the PZT sensor array and recorded by the digitizers with a sampling rate of 10 MHz.

### 4.2. Experimental Results

**Figure 5:**
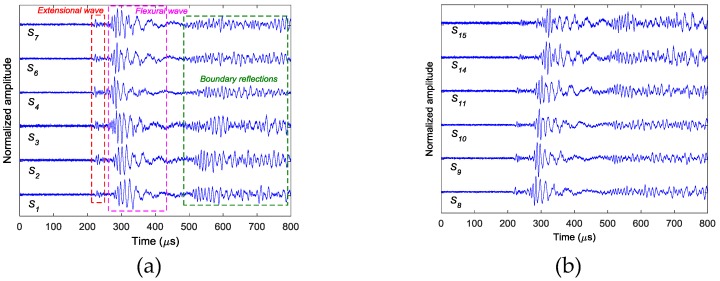
Simulated AE wave signals in case I: (**a**) sensors *S*_1_–*S*_7_ (**b**) sensors *S*_8_–*S*_15._

**Figure 6:**
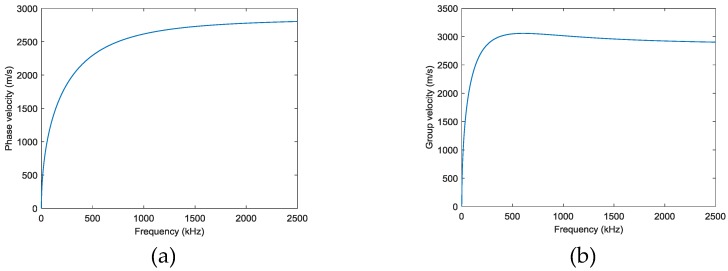
Dispersion curves of fundamental flexural wave by Mindlin plate theory: (**a**) phase velocity (**b**) group velocity.

In the experiments, three case studies are performed, i.e. (I) single damage event at (190, 250) mm simulated by exciting actuator *A*_1_; (II) single damage event at (295, 300) mm simulated by exciting actuator *A*_2_; and (III) two damage events at (190, 250) mm and (295, 300) mm simulated by simultaneously exciting both actuators of *A*_1_ and *A*_2_. Figure 5 shows the AE wave signals at 12 sensor positions when *A*_1_ is excited in case I. Since only the time information is used to localize the damage, all the signals are normalized by their maximums. Because the PZT actuators are surface mounted, both fundamental extensional wave (S0 mode Lamb wave) and fundamental flexural wave (A0 mode Lamb wave) would be excited in the frequency range of the impulse excitation. As labeled in Figure 5a, the velocity of the fundamental extensional wave is faster than the fundamental flexural wave, for each sensor response, the first arrived wave packet with relatively small amplitude is the fundamental extensional wave and the second wave packet with relatively large amplitude is the fundamental flexural wave. The wave packets after 500 µs are mainly the boundary reflections. Due to the small amplitudes, in this study, the fundamental extensional wave is neglected and only the dispersion relationships of the fundamental flexural wave are considered in the reverse-time f-k migration. Figure 6 shows the phase velocity dispersion curve and the group velocity dispersion curve of the fundamental flexural wave calculated by Mindlin plate theory [31]. These two dispersion curves are embedded in the reverse-time f-k migration algorithm to consider the dispersive properties of the AE waves.

**Figure 7:**
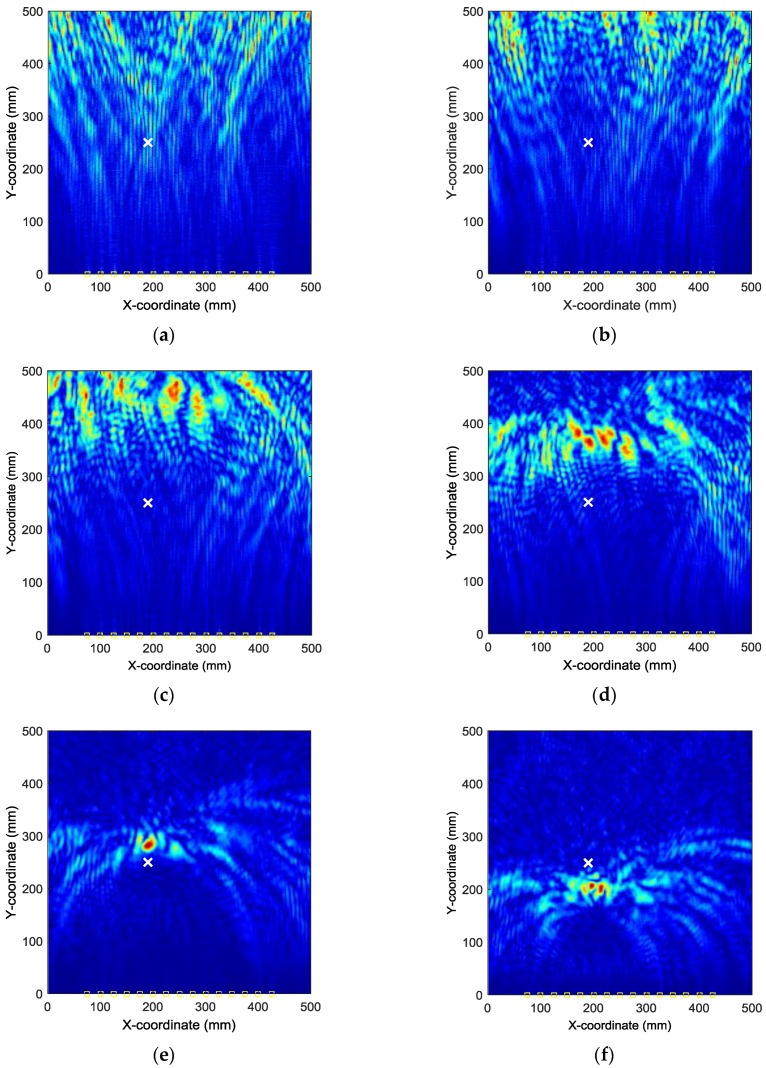
Damage images with different assumed occurrence times in case I: (**a**) 0 µs (**b**) 40 µs (**c**) 80 µs (**d**) 120 µs (**e**) 160 µs (**f**) 200 µs.

**Figure 8:**
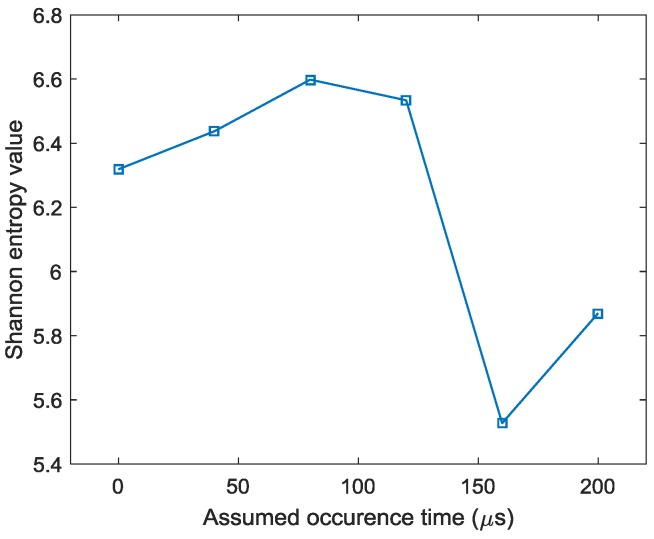
Shannon entropy values of the images with different assumed occurrence times.

By applying the reverse-time f-k migration described in Section 2 to the AE wave signals, the waves propagate back to show the spatial information about the locations of the damage events in the spatial domain. However, as it can be found from Figure 5 that the occurrence time of the damage events cannot be determined from the wave trace, it is a problem to know which length of the AE wave signals should be used to perform migration. In this study, from Figure 5, it can be seen that the damage event occurs between 0 and about 230 µs where fundamental extensional waves appear. A series of images can be produced by the AE wave signals with different assumed occurrence times. Figure 7 shows the produced damage images using reverse-time f-k migration with different assumed occurrence times in an interval of 40 µs in case I. From the figures, it can be clearly seen that the reverse-time f-k migration have successfully back-propagated the AE waves recorded by the sensor array toward its damage source (the actual simulated damage location is labeled by a ‘X’ mark for reference). But AE waves with different lengths focus to different locations as shown in Figure 7e,f, or sometimes they cannot produce a focused image as shown in Figure 7a–d. Figure 8 shows the variation of Shannon entropy values of the images shown in Figure 7. The entropy values are calculated with an image area of 500 mm × 500 mm. It can be seen that the smaller the Shannon entropy is, the clearer of the image and the closer the AE waves focus to the actual damage location is.

**Figure 9:**
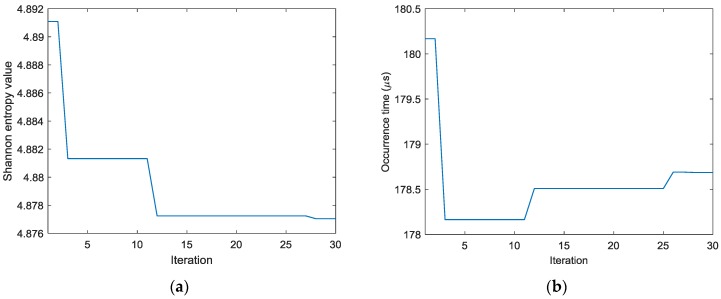
Evolution curves of results by ABC algorithm in case I (**a**) Shannon entropy value (**b**) occurrence time.

**Figure 10:**
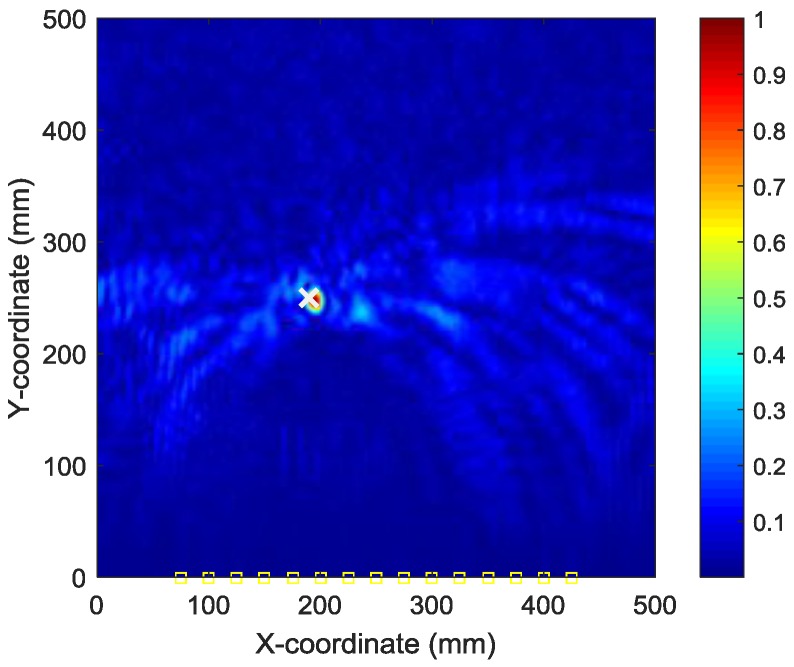
Optimal image for identification of damage source in case I.

To find the optimal image with the identified damage locations and occurrence time mostly approximating the actual ones, the ABC algorithm combined with the optimal criterion using minimum Shannon entropy described in Section 3 is applied. The number of populations in the ABC algorithm is set as 20 and the initial values of the occurrence time are randomly generated between 30 µs and 230 µs. Figure 9 shows the objective value (Shannon entropy value) evolution curve and the occurrence time evolution curve in ABC algorithm for case I. It can be found from Figure 9 that the entropy value reaches an optimal minimum with identified occurrence time of 178.7 µs (the actual one is 160 µs) and the Shannon entropy value of the optimal image is 4.877. Figure 10 shows the optimal damage image with the identified damage location indicated by the highest pixel value in case I. The actual simulated damage location is also highlighted by a ‘X’ mark for the purpose of comparison. From the figure, it can be clearly seen that combined with the optimal image criterion, the reverse-time f-k migration successfully reversed the AE waves back to its damage source. The identified location with coordinates of (197.5, 241.7) mm agrees very well with the actual one; the deviation is less than 11.2 mm (about 2.2% compared to the side length of 500 mm of the image area), demonstrating that the proposed method has the capability of imaging single damage event. The error between the identified and the actual occurrence time is because of the uncertainty of the material properties and the finite size of the actuators and sensors (they are not ideal points as assumed in the algorithms).

**Figure 11:**
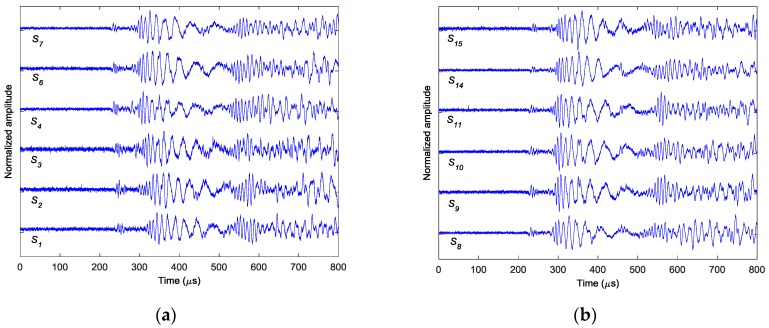
Simulated AE wave signals in case II: (**a**) sensors *S*_1_–*S*_7_ (**b**) sensors *S*_8_–*S*_15._

**Figure 12:**
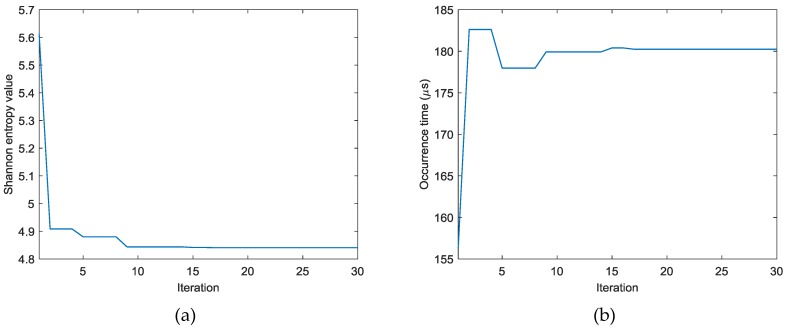
Evolution curves of results by ABC algorithm in case II: (**a**) Shannon entropy value (**b**) Occurrence time.

**Figure 13:**
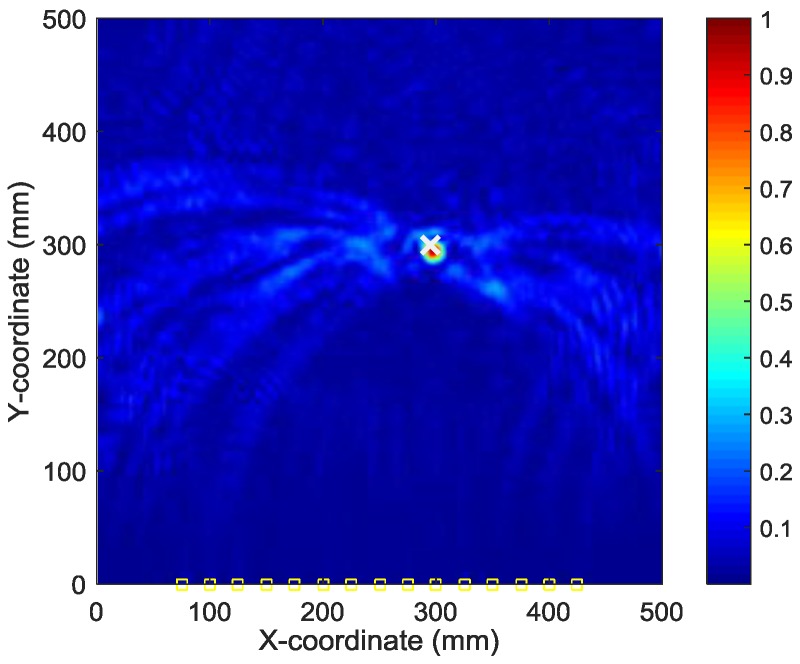
Optimal image for identification of damage source in case II.

Similar results can be obtained for cases II and III. Figure 11 shows the AE wave signals at 12 sensor positions when A_2_ is excited in case II. These AE wave signals are similar to those in case I. The same processing procedure is applied to these AE wave signals. Figure 12 shows the objective value (Shannon entropy value) evolution curve and the occurrence time evolution curve in ABC algorithm for case II. It can be found from Figure 12 that the entropy value reaches an optimal minimum with identified occurrence time of 180.2 µs and the Shannon entropy value of the optimal image is 4.841. Figure 13 shows the optimal damage image with the identified damage location indicated by the highest pixel value in case II. The identified location with coordinates of (300.0, 297.3) mm agrees very well with the actual one; the deviation is less than 5.7 mm (about 1.2% compared to the side length of 500 mm of the image area).

**Figure 14:**
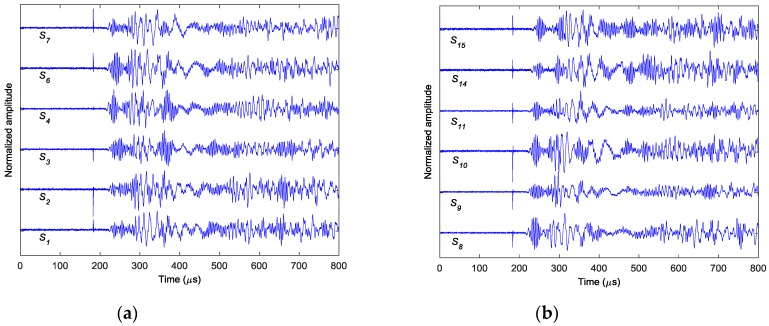
Simulated AE wave signals in case III: (**a**) sensors *S*_1_-*S*_7_ (**b**) sensors *S*_8_–*S*_15_.

**Figure 15:**
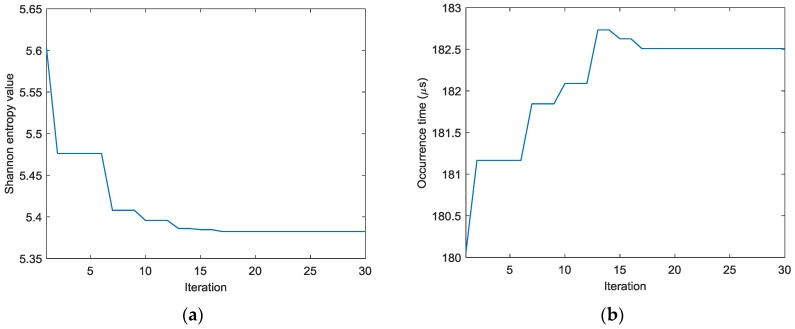
Evolution curves of results by ABC algorithm in case III: (**a**) Shannon entropy value (**b**) occurrence time.

**Figure 16:**
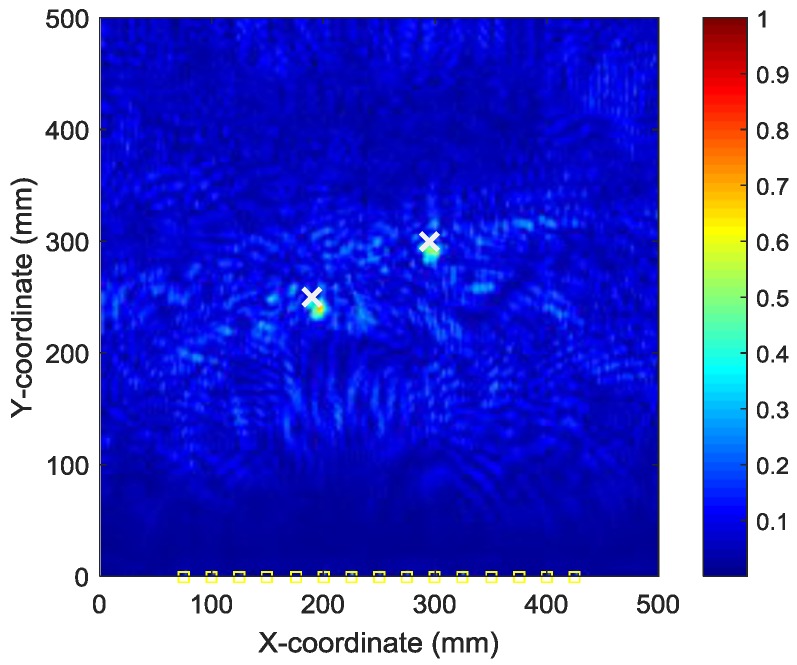
Optimal image for identification of damage sources in case III.

Figure 14 shows the AE wave signals at 12 sensor positions when both actuators of A_1_ and A_2_ are simultaneously excited in case III. This case is more complicated than the previous ones. As illustrated in Figure 14, the AE waves generated by two AE sources are totally mixed up. It is difficult for traditional signal processing-based methods to separate and discriminate them. However, as demonstrated in the following part, by the reverse-time migration, the AE waves would be separated and back-propagate to their sources. Figure 15 shows the objective value (Shannon entropy value) evolution curve and the occurrence time evolution curve in ABC algorithm for case III. As can be seen in Figure 15, the entropy value reaches an optimal minimum with an identified occurrence time of 182.5 µs and the Shannon entropy value of the optimal image is 5.382. Figure 16 shows the optimal damage image with the identified damage locations indicated by the two peak pixel values in case III. The identified locations with coordinates of (197.5, 239.1) mm and (302.5, 289.4) mm agree very well with the actual ones; the deviations are less than 13.4 mm and 13.0 (about 2.7% and 2.6% compared to the side length of 500 mm of the monitoring area), demonstrating that the proposed method has the capability of imaging multiple simultaneous damage events.

As aforementioned in Section 2, one advantage of the reverse-time f-k migration is its computational efficiency. For the experimental cases presented, the reverse-time f-k migration itself only needs less than 4 seconds to generate an image for one processing on a computer with a 4-core 3.5 GHz Xeon CPU and 8 GB memories. However, like other heuristic algorithms, the ABC algorithm takes more than 1000 seconds to perform evolution to find the optimum occurrence time, which is the main limitation of the proposed method.

### 4.3. Parametric Studies

**Figure 17:**
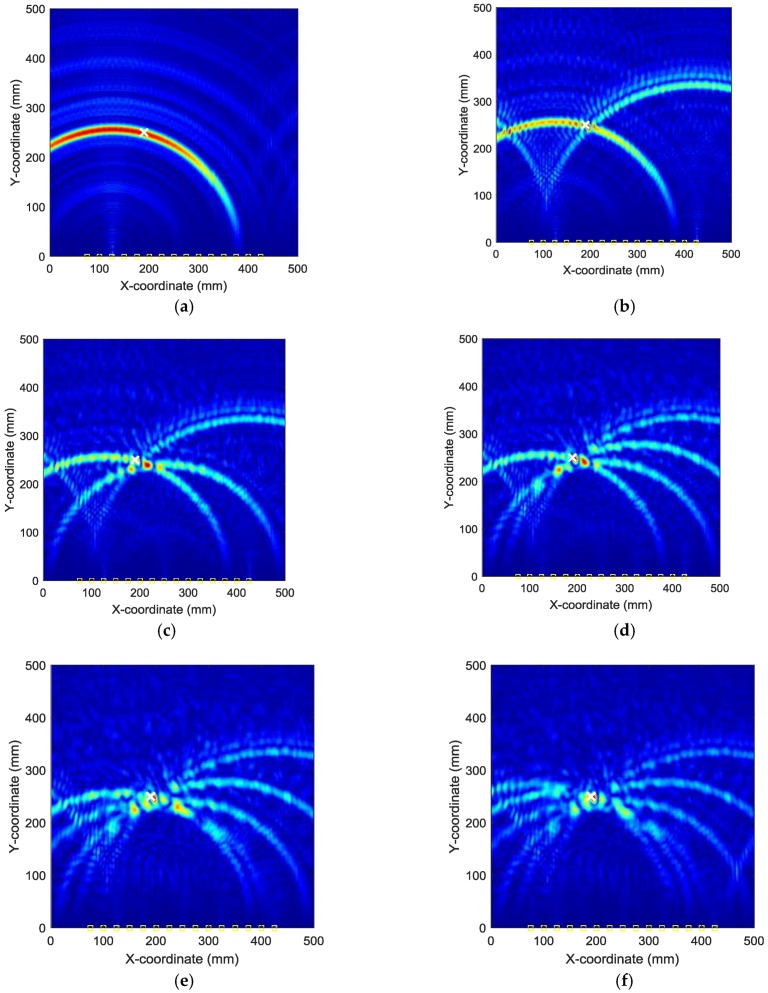
Images generated by f-k migration with signals from different number of sensors for case I: (**a**) one sensor (S_3_) (**b**) two sensors (S_3_, S_15_) (**c**) three sensors (S_3_, S_8_, S_15_) (**d**) four sensors (S_3_, S_8_, S_11_, S_15_) (**e**) five sensors (S_3_, S_5_, S_8_, S_11_, S_15_) (**f**) six sensors (S_1_, S_3_, S_5_, S_8_, S_11_, S_15_).

**Figure 18:**
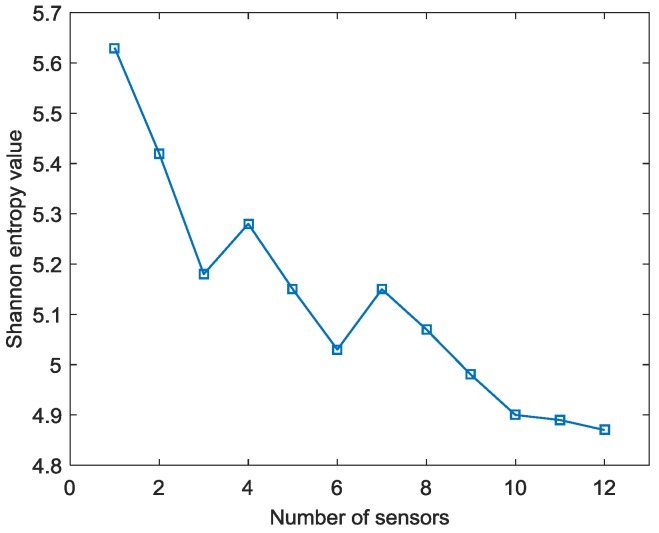
Entropy values of images generated by f-k migration with signals from different number of sensors for case I.

To further explore the proposed method, firstly the effect of sensor number on the imaging results has been studied. Taking case I as an example, Figure 17 shows images generated by f-k migration with AE wave signals collected from different number of sensors. For generating these images, the identified occurrence time of 178.7 µs is taken as the start point of the AE wave signals. It can be seen from the figures that, when only one sensor’s signal is back-propagated and imaged by f-k migration, it forms a circle with the sensor as its center on the image (as shown in Figure 17a); when signals from two sensors are employed, the intersection point of the two circles is considered as the identified AE source location (as shown in Figure 17b). The more sensors are used, the clearer the intersection point is (as shown in Figure 17c–f) (this means that it is easier to identify the damage locations from the image generated by f-k migration). Figure 18 shows the variation of entropy values of images with the number of sensors used for providing AE wave data for migration for case I. From the trend of the curve, it can be seen that in general, with the increase of sensors, the entropy value decreases, meaning the obtained image has sharper peaks near the actual source locations.

**Figure 19:**
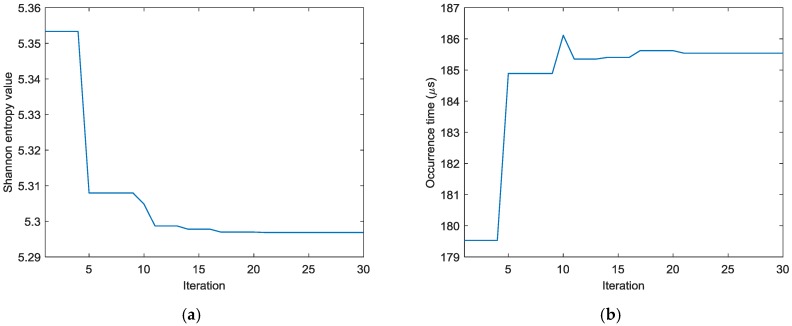
Evolution curves of results by ABC algorithm for consequent damage events with delay of 5 µs with synthetic data: (**a**) Shannon entropy value (**b**) Occurrence time.

**Figure 20:**
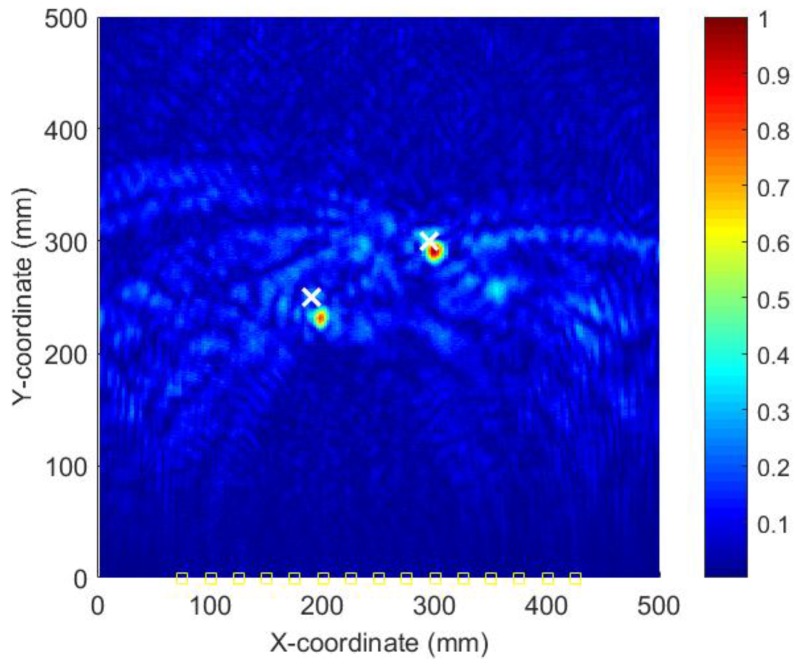
Optimal image for identification of consequent damage sources with delay of 5 µs with synthetic data.

**Figure 21:**
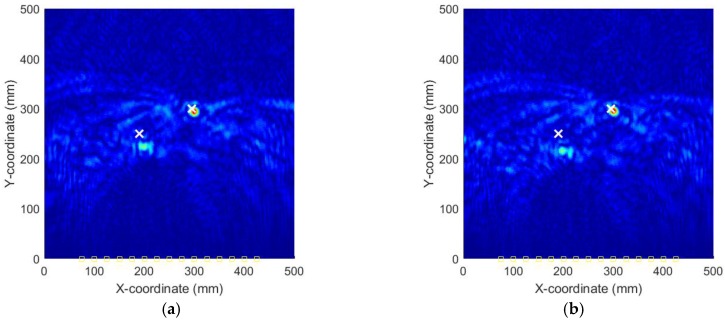

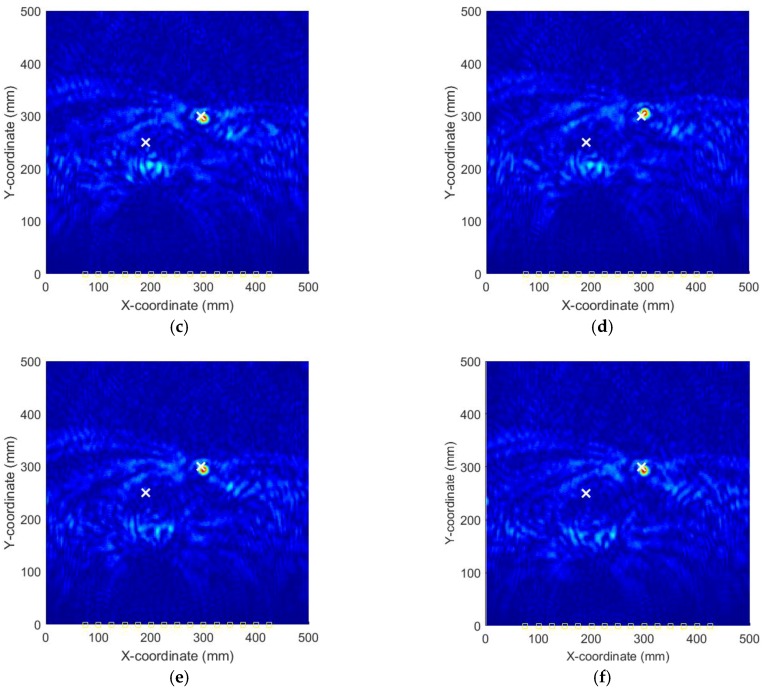
Optimal image for identification of consequent damage sources with different delays with synthetic data: (**a**) 10 µs delay (**b**) 15 µs delay (**c**) 20 µs delay (**d**) 25 µs delay (**e**) 30 µs delay (**f**) 35 µs delay.

Secondly the effect of delay between two consequent AE events on the identification results also has been studied. The previous experimental results on case III have already demonstrated that the proposed method can identify the locations of multiple simultaneous AE events. However, in reality it is unlikely to have two AE events occurring at exactly the same time. Here the AE signals from case I and case II are mixed to simulate the synthetic responses by two AE events with few delays (since the excitation forces applied by the actuators are very small, the system responses can be considered as linear and additive). In the following studies, the AE wave signals from simulated damage event at (190, 250) mm are exactly the same as those in case I and the AE wave signals from simulated damage event at (295, 300) mm in case II are delayed with specific times and added to the AE wave signals from case I to form the synthetic data (this means the AE event in case I occurs first and the AE event in case II occurs later). The synthetic data is then fed to the proposed algorithm to identify the damage locations. All the parameter settings for the algorithms are the same as those in Section 4.2. Figure 19 shows the objective value (Shannon entropy value) evolution curve and the occurrence time evolution curve in ABC algorithm for two consequent damage events with delay of 5 µs. It can be found from Figure 19 that the entropy value reaches an optimal minimum with identified occurrence time of 185.5 µs (close to the occurrence time of the second event) and the Shannon entropy value of the optimal image is 5.297. Figure 20 shows the optimal damage image with the identified damage locations indicated by the two peak pixel values for two consequent damage sources with delay of 5 µs. The coordinates of the identified locations are (197.5, 231.1) mm and (300.2, 292.0) mm and the deviations between the identified and actual locations are less than 20.5 mm and 9.5 (about 4.1% and 1.9% compared to the side length of 500 mm of the monitoring area). Figure 21 shows the produced damage images for identification of two consequent damage sources with different delays (the interval of delay times is 5 µs). It can be seen from Figure 19, Figure 20 and Figure 21, with the proposed method, one AE source can be always imaged clearly (in these results, it is the second event) and when the delays between two consequent AE events are small (less than 20 µs in Figure 21), the other AE source can be recognized although the deviations between the identified locations and the actual one increase; otherwise the images for the other AE source are very blur, it may not be recognized and its location cannot be identified just from the image.

## 5. Conclusions

This study proposes a new method for identifying the locations of AE sources in plate-like structure from the view of image processing. Reverse-time f-k migration imaging algorithm is employed to produce images containing the AE sources information and an ABC algorithm combined with an optimal criterion using minimum Shannon entropy is employed to determine the image with the identified AE source locations and occurrence time that mostly approximate the actual ones.

Experimental studies on AE waves generated by PZT actuators-simulated damage events have been performed to verify the proposed optimal image-based method. The results have shown that, with combination of a heuristic ABC optimization algorithm, the minimum Shannon entropy provides a good criterion to determine the optimal images. The identified damage locations indicated by the peaks of pixel values of the re-focused images agree the true ones very well, demonstrating that the proposed method has the capability of localizing not only single damage event but also multiple simultaneous damage events and provides a versatile tool for structural health monitoring of plate-like structures. In the future, further study should be performed to improve the computational efficiency of the overall algorithm to meet the requirement of real-time or near real-time monitoring and to consider multiple damage events occurred at different times.
